# Comparison of localization and release of multivesicular bodies and secretory granules in islet cells: Dysregulation during type‐2 diabetes

**DOI:** 10.1002/jex2.70014

**Published:** 2024-11-29

**Authors:** Priyadarshini Veerabhadraswamy, Kiran Lata, Sristi Dey, Prajakta Belekar, Lakshmi Kothegala, Vidya Mangala Prasad, Nikhil R. Gandasi

**Affiliations:** ^1^ Cell Metabolism Lab (GA‐08), Department of Developmental Biology and Genetics (DBG) Indian Institute of Science (IISc) Bengaluru India; ^2^ Molecular Biophysics Unit Indian Institute of Science Bengaluru Karnataka India; ^3^ Unit of Metabolic Physiology, Institute of Neuroscience and Physiology University of Gothenburg Gothenburg Sweden; ^4^ Center for Infectious Disease Research Indian Institute of Science Bengaluru Karnataka India; ^5^ Department of Medical Cell Biology Uppsala University Uppsala Sweden

**Keywords:** Pancreatic beta-cells, Exocytosis, Membrane trafficking, Metabolism

## Abstract

Multivesicular bodies (MVBs) are vesicles of endosomal origin containing intraluminal vesicles, which upon fusion with plasma membrane, secrete exosomes. They play a significant role in the physiology and pathology of type‐2 diabetes (T2D) due to disrupted intercellular communication. The role of MVBs and their influence on insulin secretory granules (ISGs) of β‐cells or their characterization is yet to be uncovered. In our study, we compared MVBs to largely well‐characterized ISGs in β‐cells. This study compares the density, localization, and exocytosis of CD63+ compartments (CD63+c) with NPY labelled ISGs (NISGs) in β‐cells. For this, tetraspanin CD63 was exploited to majorly label MVBs in β‐cells. These labels preserve the structural integrity of labelled compartments and mostly do not localize with other endo‐lysosomal compartments. This study showed that the β‐cells have a significantly higher density of NISGs than CD63+c. CD63+c and NISGs are spatially localized apart within β‐cells. The proteins that localize with CD63+c are different from the ones that localize with NISGs. Exocytosis of NISGs occurs at the periphery of the β‐cells and takes significantly less time when compared to the release of CD63+c, which is non‐peripheral and takes a longer duration. Mechanistically, the availability of CD63+c for exocytosis was assessed and found that an equilibrium is maintained between docking and undocking states at the plasma membrane. Although there are a high number of short‐term residing, visiting CD63+c at the plasma membrane, the availability of CD63+c for exocytosis is maintained due to docking and undocking states. Further, a significant reduction in the density of NISGs and CD63+c was observed in β‐cells isolated from T2D donors compared to healthy counterparts. Studying the effect of MVBs on insulin secretion in physiological and T2D conditions has huge potential. This study provides a strong basis to open new avenues for such future studies.

## 1 INTRODUCTION

Type‐2 diabetes (T2D) is a major metabolic disorder with increasing incidence and prevalence worldwide to the epidemic level being the ninth major cause of death worldwide (Zheng et al., 2018). The International Diabetes Federation has estimated the global prevalence of diabetes to be 9.3% (463 million people) in 2019, and it is estimated to rise to 10.2% (578 million) by 2030 and 10.9% (700 million) by 2045. About 50.1% of people with diabetes worldwide are undiagnosed (Makam et al., 2022; Saeedi et al., 2019).

Dysfunction in the secretion of islet hormones is one of the first signs of T2D. Within the islets, insulin secreting‐β, glucagon secreting‐α, and somatostatin secreting δ‐cell's viability and normal functioning are affected during human T2D (Fu et al., 2019; Kothegala et al., 2023; Omar‐Hmeadi et al., 2020; Thurmond & Gaisano, 2020). In pancreatic β‐cells, insulin is stored in membrane‐bound secretory granules called insulin secretory granules (ISGs) (Rorsman & Renström, 2003). Secretion of insulin into the bloodstream is essential for maintaining glucose homeostasis. Healthy β‐cells secrete extracellular vesicles (EVs) limiting the formation of islet amyloids thus, helping their survival. During diabetes, limited secretion of EVs is observed, correlating with the formation of islet amyloids leading to β‐cell death (Ribeiro et al., 2017). β‐cell‐derived exosomes were found to improve glucose tolerance and increase insulin content in mice with abnormal glucose tolerance (Sun et al., 2019).

Exosomes (nanosized vesicles, 35–200 nm), a type of extracellular vesicles, are released from multivesicular bodies (MVBs) (Kowal et al., 2014; Raposo & Stoorvogel, 2013; Stoorvogel et al., 2002; Welsh et al., 2024). In endocytic pathways, early endosomes mature into late endosomes and inward budding of limiting membrane of maturing/late endosomes form MVBs. MVBs contain intraluminal vesicles released as exosomes to the extracellular milieu upon the fusion of MVBs with the plasma membrane (Kalluri & LeBleu, 2020; Simons & Raposo, 2009; Stoorvogel et al., 2002). These exosomes carry cargo which includes DNA, proteins, lipids, mRNA, microRNA, and long noncoding RNA (lncRNA) (Abels & Breakefield, 2016; Colombo et al., 2014; Van Niel et al., 2018; Zhang et al., 2015). The cargo of the exosomes acts as messengers and is taken up by the cells in an autocrine and paracrine manner, therefore mediating cell‐cell communication (Février & Raposo, 2004; Huang‐Doran et al., 2017; Mathivanan et al., 2010; Meldolesi, 2018; Simons & Raposo, 2009; Tkach & Théry, 2016).

Exosomes are the widely studied, major EV type secreted from several tissue types, including the pancreas, adipose tissue, liver, immunocytes, and skeletal muscles (Chidester et al., 2020; Flaherty et al., 2019; Garcia‐Martin et al., 2022; Mei et al., 2022, Mytidou et al., 2021; Sato et al., 2016; Théry et al., 2009). In β‐cell derived EVs, the cargo was found to be altered in the case of T2D, thus, modulating insulin signalling and influencing disease development (Freeman et al., 2018). Increased miR‐29 and decreased miR‐26a in β‐cell derived EVs lead to impaired insulin signalling in neighbouring cells and induce chronic low‐grade inflammation via macrophages and monocytes in circulation (Sun et al., 2021). EVs with altered miRNA (reduced miR‐26a and NCDase, increased miR‐375‐3p and miR‐21‐5p) induce apoptotic signals in recipient β‐cells in a paracrine manner leading to cell death and dysfunction, causing diabetes‐related pathological changes (Liu et al., 2022).

MVBs and ISGs are two different vesicle types of β‐cells that fuse with the plasma membrane leading to exocytosis. ISGs are large dense core vesicles (LDCVs) of pancreatic β‐cells that store and secrete insulin. Secretion and trafficking of ISGs have been extensively studied using confocal fluorescence microscopy and total internal reflection fluorescence microscopy (TIRF) by utilizing Neuropeptide Y (NPY) as a large dense core vesicle marker (Barg et al., 2010; Gandasi et al., 2018, 2017; Omar‐Hmeadi & Idevall‐Hagren, 2021). NPY, on expression in the pancreatic β‐cells, labels the insulin secretory granules. The marker has been used extensively to study the availability of insulin granules for sustained secretion of insulin in β‐cells. The number is maintained due to the docking and undocking of insulin granules to the plasma membrane (Gandasi & Barg, 2014). Such secretion and trafficking studies of MVBs are limited in secretory cells like pancreatic β‐cells. Tetraspanin labels used to label MVBs in other cell types have opened new avenues to understanding MVB trafficking and secretion (Mahmood et al., 2023; Mathieu et al., 2021; Sung et al., 2020; Verweij et al., 2018). Tetraspanin CD63 is crucial for exosome biogenesis and cargo sorting into MVBs (Andreu & Yáñez‐Mó, 2014). The tetraspanin CD63 is present on endosomes, and plasma membrane and is enriched on intraluminal vesicles and exosomes (Escola et al., 1998). Their usage as biomarkers for MVBs in HeLa cells shows no changes in normal trafficking and secretion of MVBs (Sung et al., 2020; Verweij et al., 2018). The kinetics of MVB release and arrival to the membrane in non‐secretory cell types has provided insights into the behaviour of MVBs (Mahmood et al., 2023). The availability for MVB release maintained by a constant number of MVBs at the plasma membrane remains unexplored. Also, their importance in pancreatic β‐cells with regard to metabolic disorders has not been well studied. To understand this, tetraspanin CD63 was exploited for labelling MVBs in β‐cells to visualize the trafficking and exocytosis of these vesicles. The tetraspanin CD63 labels the MVBs by preserving their structure and mostly do not localize with other endo‐lysosomal compartments. Further, this study investigated if the trafficking and fusion of CD63+ compartments (CD63+c) are comparable to those of NPY labelled ISGs (NISGs) by using imaging techniques. A secretory cell type such as pancreatic β‐cells, where the correlation between ISGs and MVBs (source of exosomes) could be visualized was used. This study compares NISGs and CD63+c with respect to their density, localization, and exocytosis in physiological conditions. The cycle of events of CD63+c were further studied to understand the availability of these vesicles at the plasma membrane for release. Surprisingly, during pathological conditions such as T2D, the density of not only NISGs but also CD63+c was decreased. This showed that apart from insulin secretion, even survivability of islet cells was compromised during T2D. This study will open up avenues for a better understanding of how ISGs and MVBs impact each other's function and their effect on maintaining glucose homeostasis.

## 2 MATERIALS AND METHODS

### 2.1 Cells

INS‐1 832/13 cells (a kind gift from Prof. Sebastian Barg's lab, Uppsala, Sweden) were cultured in RPMI 1640 (Invitrogen, 21870‐076) supplemented with 10% foetal bovine serum (FBS, Invitrogen, A52567‐01), streptomycin (100 µg/mL), penicillin (100 µg/mL), sodium pyruvate (1 mM), l‐glutamine (2 mM) and 2‐mercaptoethanol (50 µM). MIN6 cells (a kind gift from Dr. Mostafa Bakthi's lab, Munich, Germany) were cultured in DMEM (Invitrogen, 11995‐065) media supplemented with 15% foetal bovine serum (FBS), HEPES (10 mM), streptomycin (100 µg/mL), penicillin (100 µg/mL), and 2‐mercaptoethanol (50 µM).

For transient transfection and adenovirus transduction, cells were cultured on 22‐mm poly‐l‐lysine‐coated coverslips in 6‐well plates. Transient transfections were performed on coverslips in 100 µL OptiMEM (Invitrogen, 31985‐070) using 0.5 µL Lipofectamine 2000 (Invitrogen, 11668019) or jetPRIME (Polyplus, 101000015) with 0.5 µg plasmid DNA. The reaction was terminated after 3−6 h, and imaging was performed 24−48 h after transfection. For adenovirus transduction, cells were transduced with purified adenovirus encoding mCherry‐CD63 at a 1:100 MOI (10^7^ PFU) for infection. Post 8 h of incubation, the media was replaced with fresh media, and imaging was performed 36 h after transduction.

### 2.2 Constructs

The constructs used in this study were pEGFP‐CD63 (obtained from addgene‐62964), mCherry‐CD63, created by replacing pEGFP by mCherry using multiple cloning sites in the pEGFP‐CD63 construct (developed by Vector biosystems Inc, USA), NPY‐mCherry (kindly provided by S. Barg) and syntaxin 1A‐EGFP (kindly provided by W. Almers).

### 2.3 Immunostaining

INS‐1 832/13 cells were cultured on 22‐mm poly‐l‐lysine‐coated coverslips in 6‐well plates. Cells were fixed with 4% formaldehyde after 24 h. For immunostaining with insulin antibody, cells were treated with 200 µM diazoxide 30 min before fixation. Cells without insulin staining were not treated with diazoxide. Cells were permeabilized with 0.25% triton‐X followed by blocking with 3% BSA for 45–60 min. A combination of primary antibodies was used for colocalization experiments: anti‐CD63 (1:50; Santa Cruz Biotechnology lnc., sc‐5275) with anti‐insulin (1:50; Cell Signalling Technology, 4590), or anti‐EEA1 (1:200; Abcam, ab109110) or anti‐syntaxin 1A (1.50; Abcam, ab272736) or anti‐LAMP2A (1:100; Abcam, ab18528). For analysing the tubulin network of the cells after adenovirus transduction, anti‐α‐tubulin mouse monoclonal antibody (1:200; Merck, CP06) was used. The cells were incubated overnight at 4°C after the addition of primary antibodies. Secondary antibodies, goat anti‐mouse IgG H&L conjugated with Alexa Fluor 488 (1:200; Abcam, ab150113) and goat anti‐rabbit IgG H&L conjugated with Alexa Fluor 594 (1:200; Abcam, ab150080) were incubated for 2 h at room temperature in the dark. Cell nuclei were then counterstained with Hoechst 33342 (1:1000; Invitrogen, H3570). Imaging was performed using confocal microscopy, and the images were taken with 0.3 µm or 0.6 µm z‐sections.

### 2.4 Small extracellular vesicles (sEVs) isolation

INS‐1 832/13 cells were freshly cultured on T75 flasks for the isolation of (A) unlabelled, and fluorescently labelled sEVs via (B) transient transfection with EGFP‐CD63 or (C) transduction with adenovirus mCherry‐CD63. After 8 h of transfection or adenovirus transduction, the media was replaced with fresh media containing exosome‐depleted FBS and incubated for 36 h for all conditions (A–C). Exosome depletion from FBS was carried out by ultracentrifugation (Ti70 rotor, Optima XPN‐100 Ultracentrifuge, Beckman Coulter) at 100,000 × *g*, 4°C overnight. For sEVs isolation, conditioned culture media from cells was collected and differential ultracentrifugation was performed as described by Théry et al. (2006). Briefly, 45 mL of conditioned media from each condition (A–C) was centrifuged at 300 × *g* for 10 min, 2000 × *g* for 10 min, and 10,000 × *g* for 30 min to remove dead cells and cell debris. The supernatant was collected and centrifuged at 100,000 × *g* (Type 45 Ti rotor, Optima XPN‐100 Ultracentrifuge, Beckman Coulter) for 70 min at 4°C. The pellet obtained was resuspended in 1X PBS and centrifuged at 100,000 × *g* for 70 min at 4°C. The pellet was resuspended in 50 µL of 1X PBS and stored at −80°C. The isolated sEVs were used for negative staining to visualize in transmission electron microscope.

### 2.5 Transmission electron microscopy (TEM) analysis

Isolated sEVs were loaded onto a pure Carbon Cu 300 mesh grid (TedPella Inc., 01843‐F) that was glow discharged for 45 s at 20 mA current in the glow discharge system (PELCOeasiGlow, TedPella). After 2–3 min, excess sample was blotted off using blotting paper (Whatman, 1001 125). Subsequently, the grid was stained with 1% uranyl acetate solution for 30 s and blotted off. This step was repeated one more time to ensure proper negative staining. The grid was left for drying at room temperature for 5 min and then inserted into a 120 kV Talos 120C transmission electron microscope (Thermo Fisher Scientific) for imaging. The grids were imaged at 28000X magnification equivalent to 4.99 Å per pixel. A total of 29–37 micrographs were collected for the (A) unlabelled sEVs, (B) EGFP‐CD63 labelled and (C) mCherry‐CD63 adenovirus transduced sEVs. The number and diameters of sEVs were measured from the micrographs for each sample. Analysis and measurements were carried out using IMOD 4.10.15 (Kremer et al., 1996).

### 2.6 Islet cell isolation and transduction

Human pancreatic tissue was obtained with the informed consent of the families involved, from the Nordic Network of Clinical Transplantation (Uppsala Regional Ethics Board ethical approval 2006/348) (Gandasi et al., 2018) or the ADI Isletcore at the University of Alberta (Alberta Human Research Ethics Board ethical approval Pro00001754) (Lyon et al., 2016). Work involving human tissue complies with all applicable ethical standards for use in research, and the Gothenburg Regional Ethics Board, Sweden (098‐18) and ethical committee at the Indian Institute of Science, India (02/24.02.2023). The acquired tissue was cultured overnight and the islets were isolated (Gandasi et al., 2018). Trypsinization and plating of the isolated islets were performed to obtain single cells. The resulting single cells were maintained at 37°C and 5% CO_2_ post being cultured in CMRL 1066 medium supplemented with 5.5 mM glucose, 10% FBS, 2 mM L‐glutamine, and 1% penicillin‐streptomycin. Cells were plated on 22‐mm poly‐L‐lysine‐coated coverslips and incubated overnight. Adenovirus particles were added to the culture media at a concentration of 1:100 MOI (10^7^ PFU) for infection and cultured for 24–36 h before being imaged. The adenovirus particles were either admCherry‐CD63 or adNPY‐mCherry (Meur et al., 2010).

### 2.7 Solutions

Cells were imaged in a solution containing 138 mM sodium chloride (NaCl), 5.6 mM potassium chloride (KCl), 1.2 mM magnesium chloride (MgCl_2_), 2.6 mM calcium chloride (CaCl_2_), 10 mM d‐glucose, and 5 mM HEPES (pH 7.4, adjusted with 1 M sodium hydroxide (NaOH)) for density analysis of CD63+c. Buffer instead contained 10 mM glucose supplemented with 200 µM diazoxide for density analysis of NISGs.

For exocytosis of NISGs, the buffer contained 10 mM glucose and was supplemented with 2 mM forskolin and 200 µM diazoxide, a K^+^ ATP channel opener that prevents glucose‐dependent depolarization. Exocytosis was then evoked by computer‐timed local application of high K^+^ (75 mM KCl equimolarly replacing NaCl) through a pressurized glass electrode similar to those used for patch clamp experiments. Exocytosis of CD63+c was evoked by the application of 100 µM histamine in a similar way as described for the high K^+^ above.

### 2.8 Microscopy

For two‐colour TIRF imaging for colocalization, and for single‐colour, temporal imaging of fusion events, cells were imaged at 37°C using a total internal reflection fluorescence (TIRF) microscope based on an AxioObserver Z1 with a 100 ×/1.45 objective (Carl Zeiss, Jena, Germany). Excitation was from two DPSS lasers at 491 and 561 nm or individual lasers as described in individual experiments. For colocalization experiments, the emission light was chromatically separated into different areas of an EMCCD camera (Photometrics Evolve) using an image splitter (Photometrics DV2, Photometrics, Tucson, AZ, USA). The acquisition was through 16‐bit images with 0.16 µm/pixel resolution. The alignment of the two‐colour channels was corrected as previously described (Taraska et al., 2003).

For density and behavioural analysis of the CD63+c and for density analysis of NISGs, cells were imaged at 37°C using a total internal reflection fluorescence (TIRF) microscope, Nikon ECLIPSE Ti2 at 100 ms exposure. Time‐lapse images for behavioural events of CD63+c were captured with 488 excitation filter for 1 min, without delay by EMCCD camera (Andor). The acquisition was through 16‐bit images with 0.048 µm/pixel resolution.

For immunostaining experiments, imaging was done using Nikon ECLIPSE Ti2 confocal microscope at room temperature. The images were captured with 0.3 µm z‐sections for colocalization and density analysis experiments or 0.6 µm *z*‐sections for tubulin network analysis using an EMCCD camera (Andor). Excitation was performed individually using three DPSS lasers at 561, 488, and 405 nm. Images obtained were 16‐bit with 0.09 µm/pixel resolution. The top sections of the z‐stacks were later selected for analysis.

### 2.9 Image analysis

The density of the vesicles in INS‐1 832/13 cells, non‐diabetic (ND) and type‐2 diabetic (T2D) human islet cells were calculated using a script that used the built‐in ‘find maxima’ function in ImageJ (http://rsbweb.nih.gov/ij) for spot detection (Gandasi & Barg, 2014; Makam et al., 2024). This count was then normalized to the area.

Colocalization was estimated using MetaMorph software. Regions of interest (ROIs) were marked manually in green (Figure 2e) or red channel (Figure 2h) after identifying the vesicles. When these ROIs were transferred to the other channel, the centring was marked by a yes/no choice. This was based on the ROI positioning within one pixel at the centre of the previously identified ROIs. These were plotted as percentage colocalization.

Visualization of exocytosis of vesicles in the form of their fluorescence changes was performed using the image analysis software MetaMorph (Molecular Devices, Sunnyvale, CA, USA). Fluorescence changes of exocytosis events were captured during the period of the time series. During exocytosis, there is a sudden disappearance of fluorescence within a few milliseconds as visualized in the TIRF field. These changes were treated with an algorithm implemented in the MetaMorph journal. This journal reads the average pixel fluorescence of the granule in (1) a central circle (c) of 3 pixels diameter, (2) a surrounding annulus (a) with an outer diameter of 5 pixels. Granule fluorescence ∆F was obtained by subtracting the circle (c) with the annulus value (a) (∆*F* = *c*−*a*). This value of ∆F was given as per‐pixel average for the entire 3 pixel circle; this covers the entire size of the puncta. ∆*F* values of the vesicles were plotted against time (s) (Barg et al., 2010).

The time course of release from the fusion site is measured using two approaches. (a) Visual tracking of the puncta throughout the movie. (b) Measuring the intensity within the ROI in an unbiased way. The change in fluorescence was calculated by Zheng et al. (2018). Marking the puncta as regions of interest based on observable fluorescence change, by confirming the same with the ∆*F* versus time graph. The fusion time is taken as time ‘0’ at the point of bright/higher fluorescence just before the significant loss of fluorescence (Saeedi et al., 2019). This was also done by averaging the ∆*F* values of the marked ROI before and after the event, taken as mentioned in Zheng et al. (2018). Makam et al. (2022). All the selected points were passed through a function where the point that is bigger than the standard deviation of previous data points was evaluated, to see if there is a drop bigger than the standard deviation. The point ‘0’ of all the ROIs is then aligned to obtain an average ∆*F* versus time graph for all the ROIs. Similarly, the release endpoint is selected by observing both events and their corresponding ∆*F* versus time graphs. The average ∆*F* of ROI at the endpoint after the first observable loss of fluorescence is similar to the ∆*F* seen from some random non‐puncta ROI selected within the same cell (Alenkvist et al., 2017; Barg et al., 2010; Gandasi & Barg, 2014). The time taken for release is then measured by calculating the frame number from point 0 (time taken as 0 s) till the release endpoint as images were captured at 100 ms per frame. The events that did not start to decay or fully decay, and the events where the movie ended before the decay was complete were eliminated from the analysis to calculate the average release time. The ROIs were selected for those events that showed stable docking, followed by fusion and release at the end of the movie.

For identifying the exocytosis events as peripheral and non‐peripheral, from the border of the cells, a region of 10 pixels (1.6 µm) were marked. Events observed as fluorescence change within the region of 10 pixels (1.6 µm) from the border were considered as peripheral events and the events in the region after 10 pixels (1.6 µm) from the border towards the centre were considered non‐peripheral events.

Granules in the TIRF field were identified and marked as ROIs. Docking, Undocking, Docking and Undocking, and Visiting events were identified based on the criteria below: Docking event was defined as granules that appeared and were confined to the defined ROI till the end of the movie. The last part of the image stack was not considered to ensure a residence of at least >5.92 s (40 frames). Undocking events are docked granules identified at the start of the movie, residing for at least > 5.92 s (40 frames) before slowly disappearing or moving away from the ROI. Visitors were those granules that appeared for <5.92 s (40 frames) with a residence of at least 0.59 s at the defined ROI before disappearing or moving out of the ROI. Docking and undocking events are the granules that appeared in the defined ROI and remained docked for at least 5.92 s (40 frames) before slowly disappearing or moving away from the docked site.

Fluorescence changes of these events were calculated using an algorithm implemented in the MetaMorph journal. ∆*F* values of the individual vesicles or average ∆*F* values of vesicles were plotted against time as mentioned above. The residence time of vesicles was plotted by calculating the time spent by the vesicles in the defined ROI based on fluorescence change.

In parallel, the track mate function of Image J was used to assess the mobility of visiting and, Docking and Undocking events within a region of 0.3 µm.

The density of the vesicles in INS‐1 832/13 cells and MIN6 cells was calculated using MetaMorph software (figure S2C and S2F). ROIs were marked manually in the red or green channel after identifying the vesicles. This count was then normalized to the area of the cell.

The fluorescence intensity of the tubulin network was calculated using a script that used the built‐in ‘find maxima’ function in ImageJ (http://rsbweb.nih.gov/ij) for spot detection. The average fluorescence intensity of all the spots detected was then calculated for each cell.

### 2.10 Statistics

Data is presented as mean ± SEM unless otherwise stated.  All the other data was tested for statistical significance using Students t‐test for one‐tailed, unpaired samples, as appropriate. Significant difference is indicated by asterisks (*p < 0.05, **p < 0.01, ***p < 0.001).

## 3 RESULTS

### 3.1 Density and labelling of MVBs

**FIGURE 1:**
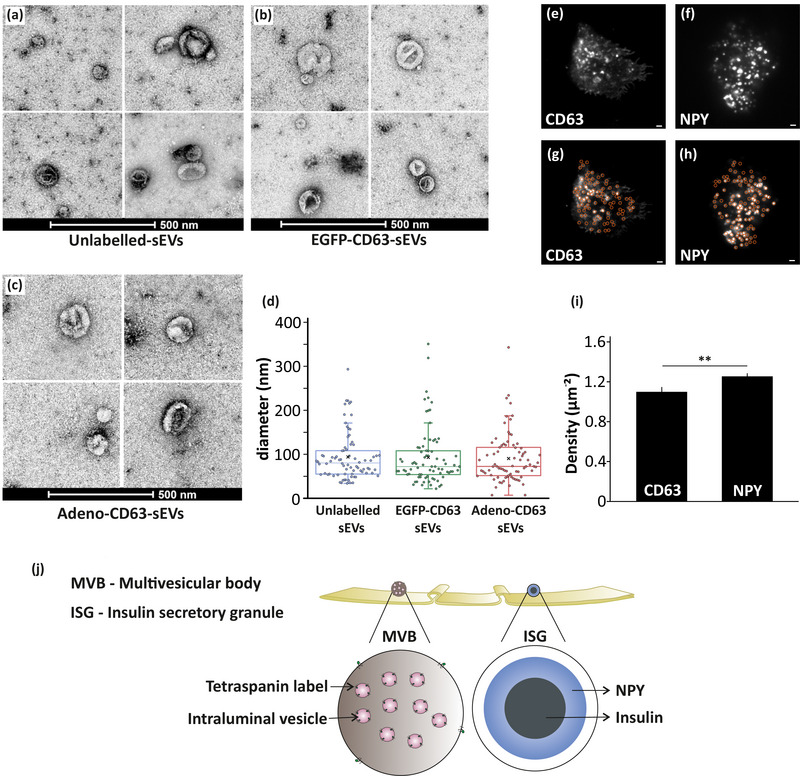
Density and labelling of multivesicular bodies (MVBs) and insulin secretory granules (ISGs). (a–c) Negative‐staining transmission electron microscopy (TEM) images of isolated sEVs: (a) Unlabelled, (b) EGFP‐CD63 transfected and (c) CD63‐mCherry adenovirus transduced. Scale bar 500 nm. (d) Comparison of diameter/size (nm) and size distribution of isolated sEVs (for images like a–c). Data are represented as box plots for *n* = 78 unlabelled, EGFP‐CD63 labelled, and adenovirus mCherry‐CD63 labelled sEVs. The box represents the interquartile range with horizontal line representing the median and X representing the mean of the data. (e) Image of a cell expressing EGFP‐CD63 labelling CD63+ compartments (TIRF microscopy). Scale bar 1 µm. (f) Image of a cell expressing NPY‐mCherry labelling ISGs (TIRF microscopy). Scale bar 1 µm. (g,h) Detection of (g) CD63+c or (h) NISGs based on the analysis described in methods. Scale bar 1 µm. (i) Density of EGFP‐CD63 and NPY‐mCherry (for images like in e,f). Data are presented as mean ± SEM for *n* = 30 cells (EGFP‐CD63) and *n* = 40 cells (NPY‐mCherry) from at least two independent experiments in each case. **p < 0.01 (details in the methods). (j) Schematic representation of tetraspanin labelled MVBs and NPY labelled ISGs.

To study MVB and ISG populations in pancreatic β‐cells, the strategies implied in previous literature for labelling MVBs (Bebelman et al., 2020; Mathieu et al., 2021; Verweij et al., 2018, 2019, 2021) and LDCVs (Barg et al., 2010; Gandasi et al., 2015) was explored. There are no studies on labelling and visualizing the exocytosis of MVBs in pancreatic β‐cells. This study exploited tetraspanin CD63 to label MVBs in β‐cells based on their ability to label MVBs in other cell types (Mahmood et al., 2023; Mathieu et al., 2021; Verweij et al., 2018). We wanted to confirm the secretion of small extracellular vesicles (sEVs) including exosomes from pancreatic β‐cells and evaluate if fluorescent labelling of CD63 via transfection or transduction affects the size and morphology of sEVs. sEVs were isolated from (A) unlabelled cells, (B) cells transfected with EGFP‐CD63 and (C) cells transduced with adenovirus mCherry‐CD63 and were observed using negative stain transmission electron microscopy (NS‐TEM). sEVs isolated from these samples were observed to have a similar spherical shape. Diameters were measured for a total of 78 sEVs from unlabelled, EGFP‐CD63 transfected, and adenovirus‐mCherry‐CD63 transduced cells. These sEVs were similar in size with an average diameter of 94.0 nm for (A) unlabelled‐sEVs, 92.8 nm for (B) EGFP‐CD63 labelled sEVs, and 90.4 nm for (C) adenovirus‐mCherry‐CD63 sEVs (Figure 1a–c). We confirmed that the fluorescent labelling of CD63 does not affect the size and morphology of the isolated sEVs (Figure 1d). We also wanted to evaluate if adenovirus transduction affects the microtubule network which is essential for the normal trafficking of MVBs within the cells. For this, immunostaining of CD63 transduced cells for α‐tubulin was followed where no significant difference between transduced and non‐transduced cells was observed (figure S1).

Further, this study exploited EGFP‐CD63 to label CD63+ compartments/MVBs of pancreatic β‐cells. When EGFP‐CD63 was overexpressed in β‐cells, punctate structures were observed near the plasma membrane of β‐cells (Figure 1e), similar to what had been observed in HeLa cells (Verweij et al., 2018). Similarly, NPY‐mCherry labelled ISGs were observed near the plasma membrane of β‐cells (Figure 1f). Fluorescent puncta observed in single cells using TIRF microscopy was quantified using an Image J Plugin “find maxima” (Figure 1g–h) (details in the methods). The density of EGFP‐CD63 labelled compartments and NPY‐mCherry (LDCV marker) labelled ISGs were normalized to the area of the cell. The density of CD63+c observed was 1.099 ± 0.047 µm^−2^ and that of NISGs was 1.254 ± 0.032 µm^−2^. The β‐cells were found to contain significantly higher density of NISGs than CD63+c (p < 0.01) (Figure 1i). Similar experiments were performed in MIN6 cells (another murine β‐cell line) to check the density of these vesicles. Immunostaining was done to compare endogenous levels of CD63 and insulin in two different pancreatic β‐cell lines INS‐1 832/13 and MIN6. In MIN6 cells, the density of insulin granules was marginally higher than CD63+c (figure S2D–F) whereas significantly higher in INS‐1 832/13 cells (figure S2A–C).

### 3.2 Localization of MVBs compared to ISGs

**FIGURE 2:**
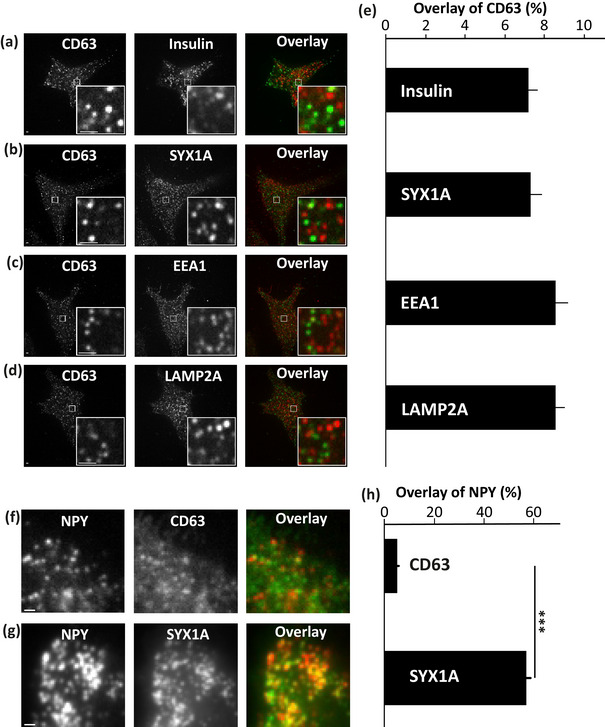
Localization of multivesicular bodies (MVBs) with other vesicle types. (a) Immunofluorescent images of endogenous CD63 in the green channel and endogenous insulin, labelling ISGs in the red channel with a corresponding image in the overlay imaged using confocal microscopy. Enlarged insets displayed to the right (bottom) measured at 8X zoom. Scale bar 1 µm. (b) Same as A for syntaxin‐1A in the red channel. (c) Same as A for EEA1 in the red channel. (d) Same as A for LAMP2A in the red channel. (e) Colocalization from images such as (a–d) calculated as described in the methods. Data are represented as mean ± SEM for *n* = 20 cells for each case from at least two independent experiments. (f) TIRF Images of a cell expressing NPY‐mCherry, labelling NISGs in the red channel with EGFP‐CD63, labelling CD63+c in the green channel with a corresponding image in the overlay. Scale bar 1 µm. (g) TIRF Images of a cell expressing NPY‐mCherry, labelling NISGs in the red channel with syntaxin 1A‐EGFP in green channel with a corresponding image in the overlay. Scale bar 1 µm. (h) Colocalization from images such as (f,g) calculated as described in the methods. Data are represented as mean ± SEM for *n* = 36 cells for each case from at least three independent experiments. ***p < 0.001.

To evaluate, if the proteins regulating the exocytosis of ISGs are utilized for MVB exocytosis, the localization of MVBs with ISGs near the plasma membrane was analysed. First, the endogenous distribution and localization of CD63 were evaluated. The localization of endogenous CD63 with markers of other vesicle types like early endosomes, lysosomes, and large dense core vesicles of pancreatic β‐cells was analysed by confocal imaging. Immunostaining of CD63 with ISG marker Insulin (Figure 2a), early endosomal marker EEA1 (Figure 2c), and lysosomal marker LAMP2A (Figure 2d) in pancreatic β‐cells showed no major colocalization. The percentage colocalization of CD63 near the plasma membrane was 7.2, 8.5, and 8.5 for ISGs, EEA1 and LAMP2A, respectively (Figure 2e).

Since, insulin secretion via the exocytosis of ISGs is the key exocytotic event in pancreatic β‐cells, the localization of MVBs with ISGs near the plasma membrane was checked to evaluate if the proteins regulating exocytosis of ISGs are utilized for MVB exocytosis as well. Cells co‐transfected with NPY‐mCherry and EGFP‐CD63 or NPY‐mCherry and syntaxin 1A‐EGFP were imaged using TIRF microscopy. Syntaxin‐1A was used as a control since it is a t‐SNARE, which facilitates the fusion of ISGs to the plasma membrane. Overlay of NPY‐mCherry with EGFP‐CD63 (Figure 2f) or syntaxin 1A‐EGFP (Figure 2g) was analysed for colocalization using MetaMorph (details in methods). The percentage colocalization of NPY‐mCherry with EGFP‐CD63 was found to be 5.0% whereas it was 57.0% for NPY‐mCherry colocalizing with syntaxin 1A‐EGFP (Figure 2h). This data suggests that NPY‐mCherry labelled ISGs are spatially apart from EGFP‐CD63 labelled compartments. These were compared to positive control NPY‐mCherry, which had a significantly higher degree (*p* < 0.001) of overlap with syntaxin 1A‐EGFP (Figure 2h). The colocalization of endogenously expressed CD63 with endogenous syntaxin‐1A (Figure 2b) was also checked by confocal imaging near the plasma membrane. The localization of CD63 with syntaxin‐1A was found to be minimal with 7.3% (Figure 2e). This study shows that the MVBs and ISGs are spatially localized apart in β‐cells and also MVBs are not localizing with syntaxin‐1A. Therefore, MVBs and ISGs cannot utilize the exocytosis machinery of each other.

### 3.3 Exocytosis of MVBs and ISGs have distinct dynamics

**FIGURE 3:**
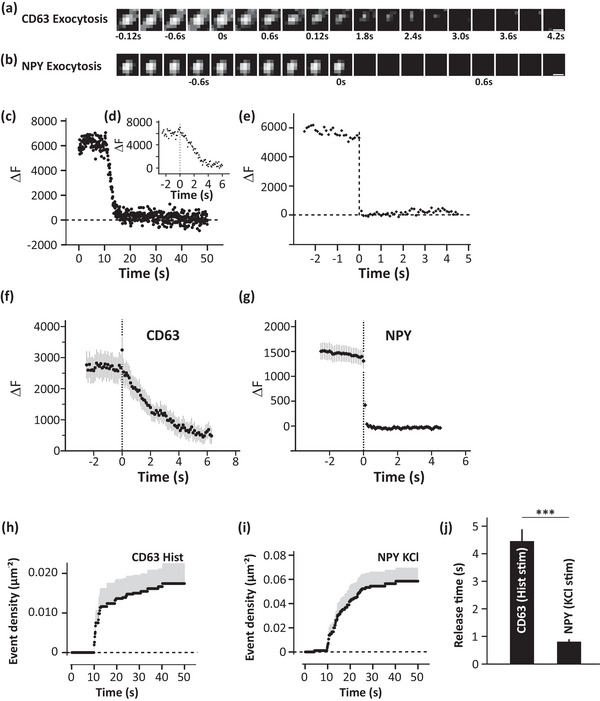
Exocytosis of MVBs and ISGs. (a) Time series strip showing the disappearance of EGFP‐CD63 from the plasma membrane due to exocytosis captured using TIRF microscope. Scale bar 0.5 µm. (b) Same as (a) for NISGs. Scale bar 0.5 µm. (c) Fluorescence from a single exocytotic event of CD63+c (EGFP‐CD63) over a period of 50 s showing the kinetics of release. Fluorescence is quantified using Δ*F* as described in the methods. (d) Same as (c) over a period of 6 s. (e) Same as (c) for a single exocytotic event of NISGs (NPY‐mCherry) over a period of 4.5 s. (f) Average fluorescence from many exocytotic events of CD63+c (EGFP‐CD63) such as (c) over time showing the dynamics of release. (g) Same as (f) for NISG (NPY‐mCherry) events. (h) Density of exocytosis events of CD63+c (EGFP‐CD63) after stimulation with histamine. The stimulation was initiated at 10 s. (i) Same as (h) for exocytotic events of NISGs (NPY‐mCherry) after stimulation with KCl. J Average release time for the exocytosis of CD63+c (EGFP‐CD63) and NISGs (NPY‐mCherry) based on kinetics plotted for events similar to the ones plotted in (d) and (e). Data are presented as mean ± SEM for *n* = 9 cells for each case. ***p < 0.001.

In this study, the exocytosis of MVBs and ISGs was visualized to compare the kinetics of fusion and release of both vesicle types in β‐cells. The INS‐1 832/13 cells transfected with EGFP‐CD63 or NPY‐mCherry were imaged, to visualize the exocytosis of MVBs and ISGs, respectively, using TIRF microscopy. Cells were stimulated for MVB and ISG release (details in the methods), where individual fluorescent puncta disappear in one frame over time. Such events were identified as release events in the series of time‐lapse images captured based on the criteria: a) disappearance of fluorescence in a single frame b) loss of fluorescence without any reappearance in the same region during the time duration of the experiment. These criteria were followed for the detection of CD63+c (EGFP‐CD63) and NISG (NPY‐mCherry) exocytosis (Figure 3a,b). The single release event of CD63+c (Figure 3c,d) and NISGs (Figure 3e) were plotted as ΔF over time with average ΔF of many such events shown in Figure 3f (EGFP‐CD63) and Figure 3g (NPY‐mCherry) (details in the methods). The average Δ*F* of fusion events of CD63+c showed an increase in the fluorescent intensity upon fusion of EGFP‐CD63 with the plasma membrane indicating the fusion.

The average release time was calculated by analysing multiple release events of CD63+c and NISGs (Figure 3j). The average release time for CD63+c was above 4 s and the same was less than 1 s for NISGs after stimulation. It is clear that MVBs take a significantly higher time for release when compared to ISGs (*p* < 0.001) (Figure 3j). A slower MVB release in pancreatic β‐cells was observed in line with the MVB release time shown by Verweij et al. (2018), in HeLa cells. A histogram based on the fusion events of CD63+c Figure 3h) and NISGs (Figure 3i) upon stimulation (stimulation starts 10 s after the beginning of the experiment, details in the methods) was plotted to compare the density of release events. Most of the release events both in the case of CD63+c and NISGs started within 10 s after stimulation (Figure 3h,i). The number of fusion events was significantly lower in the case of CD63+c (0.017 ± 0.005 µm^−2^) when compared to NISGs (0.058 ± 0.010 µm^−2^). These data indicate that MVBs and ISGs follow distinct release kinetics.

### 3.4 MVBs and ISGs have preferential site of release

**FIGURE 4:**
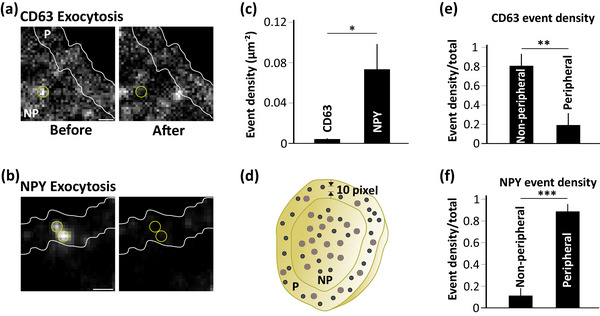
Preferential release site of MVBs and ISGs. (a) An image showing CD63+c exocytosis event, before (left) and after (right) loss of fluorescence of EGFP‐CD63 as peripheral (P, within 10 pixels from the border of the cell) and non‐peripheral (NP, at least 10 pixels away from the border of the cell) events as described in methods. Scale bar 1 µm. (b) Same as A for NISGs. Scale bar 1 µm. (c) Density of total exocytosis events of CD63+c (EGFP‐CD63) and NISGs (NPY‐mCherry). Data are represented as mean ± SEM for *n* = 8 cells for each case. *p < 0.05. (d) Schematic diagram showing the peripheral and non‐peripheral region of a cell considered for analysis. (e) Density of peripheral and non‐peripheral events as a fraction of total events observed during exocytosis of CD63+c (EGFP‐CD63). **p < 0.01. (f) Same as (e) for NISGs (NPY‐mCherry). ***p < 0.001.

The observed difference in the localization and release kinetics of MVBs and ISGs led us to look for their release site within the cell. Our previous results described that MVBs and ISGs are spaced apart in non‐stimulatory conditions (Figure 2h). Further evaluation on whether they remained apart during stimulated conditions as well was carried out. To evaluate the distribution and density of release events, the labelled cell was classified into peripheral (P, within 10 pixels from the border) and non‐peripheral regions (NP, after 10 pixels from the border towards the centre) (Figure 4d). The release events of CD63+c and NISGs were identified based on the criteria (more details in the methods) mentioned above (Figure 3a,b).

The release events identified in peripheral and non‐peripheral regions of a cell are shown as before and after exocytosis of CD63+c (EGFP‐CD63, Figure 4a) and NISGs (NPY‐mCherry, Figure 4b), respectively. The total number of release events of CD63+c and NISGs were normalized to the area of the cell to calculate the density of the distribution of events. The density of release events overall for CD63+c (0.004 µm^−2^) was significantly lower (*p* < 0.05) when compared to the NISG event density (0.073 µm^−2^) (Figure 4c). The same data represented as fusion events/min/cell shows 3.7 fusion events of CD63+c which is significantly lower as compared to 7.4 ISG fusion events. Further evaluation on, how many of the total events are localized to peripheral or non‐peripheral regions (as described previously) was done to express as a fraction of peripheral and non‐peripheral events of CD63+c and NISGs respectively. CD63+c were released preferentially from the non‐peripheral region (NP events = 0.003 µm^−2^ (3.2 events/min), fraction non‐peripheral = 0.808/total) when compared to the peripheral region (*P* events = 0.001 µm^−2^ (0.50 events/min), fraction peripheral = 0.192/total) (*p* < 0.01) (Figure 4e). On the other hand, NISGs released significantly (*p* < 0.001) more from the periphery of the cells (*P* events = 0.069 µm^−2^ (6.41 events/min), 0.888/total) when compared to the non‐peripheral release events (NP events = 0.005 µm^−2^ (1.0 events/min), 0.112/total) (Figure 4f). Therefore, MVBs release preferentially from non‐peripheral regions and ISGs from peripheral regions, indicating their distinct release site during exocytosis in β‐cells.

### 3.5 Mechanistic insights into the availability of MVBs for release

Studying the subcellular distribution of MVBs will give an idea about the availability of MVBs at the plasma membrane that secretes exosomes. Although the density of MVBs at the plasma membrane is high, only a fraction of them undergo secretion. To know, if the constant number of MVBs is maintained at the plasma membrane, we dwelled to understand the mechanistic details of how equilibrium is maintained during MVB exocytosis. Here, different MVB behavioural events near the plasma membrane were characterized. CD63+c in the TIRF field were identified and marked as regions of interest (ROI), to analyse their behaviour.

**FIGURE 5:**
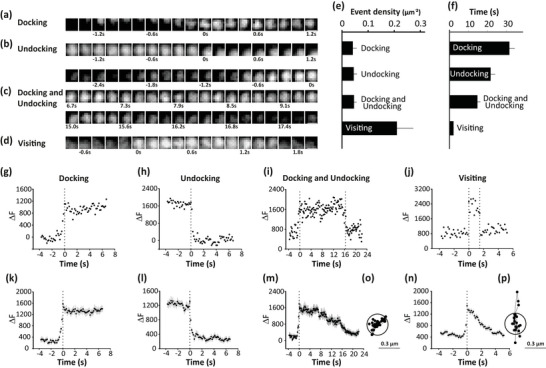
Mechanistic insights into the availability of MVBs for release. (a–d) Time series strip showing different CD63+c (EGFP‐CD63) events near the plasma membrane captured via TIRF microscopy. (a) Docking, (b) Undocking, (c) Docking and Undocking and (d) Visiting. Scale bar 0.25 µm. (e) Density of different CD63+c (EGFP‐CD63) events near the plasma membrane. Data are presented as mean ± SEM for *n* = 18 (Docking), *n* = 22 (Undocking), *n* = 21 (Docking and Undocking), and *n* = 95 (Visiting) from six cells from at least two independent experiments. (f) Residence time of different CD63+c (EGFP‐CD63) events near the plasma membrane. Data are presented as mean ± SEM for *n* = 18 (Docking), *n* = 22 (Undocking), *n* = 21 (Docking and Undocking), and *n* = 95 (Visiting) from six cells from at least two independent experiments. (g–j) Fluorescence from single CD63+c (EGFP‐CD63) events shown in (a–d) over time representing the dynamics of single event (g) Docking, (h) Undocking, (i) Docking and Undocking and (j) Visiting. (k–n) Average of fluorescence from many CD63+c (EGFP‐CD63) events such as (g–j) over time showing the dynamics of (k) Docking, (l) Undocking, (m) Docking and Undocking and (n) Visiting. *n* = 39 (Docking), *n* = 43 (Undocking), *n* = 21 (Docking and Undocking), and *n* = 95 (Visiting) from six cells from at least two independent experiments. (o) Tracking of a Docking and Undocking event showing the puncta confined to the defined ROI of 0.3 µm diameter for ∼7 s. (p) Tracking of a visiting event showing the rapid movement of puncta around the defined ROI of 0.3 µm diameter for ∼4 s.

The events that were observed include—(a) CD63+c appearing in the defined ROI in the TIRF field and stably staying within the ROI, without major movement along XY plane or Z plane till the end of the movie (residing at least for 40 frames, example in Figure 5a shows an event and fluorescence plotted as ΔF over time in Figure 5g). These events were considered docking events (dynamics plotted in Figure 5k). (b) A small population of CD63+c identified at the start of the movie stably docked in the defined ROI of the TIRF field and disappeared from the ROI by moving along the XY plane or the Z plane (after residing for at least 40 frames, for example, Figure 5b shows an event and Figure 5h shows fluorescence plotted as ΔF over time). These events were considered undocking events (dynamics plotted in Figure 5l). (c) Apart from docking, a population of CD63+c similarly appears in the defined ROI in the TIRF field but for a much shorter duration (<40 frames, example in Figure 5d shows an event and fluorescence plotted as ΔF over time in Figure 5j) and showed higher XY and Z movement even outside the defined ROI, these were considered as visiting events (dynamics plotted in Figure 5n). (d) There were instances where docking and undocking were seen within the time duration of the experiment but with similar characteristics as said in (a) and (b) (example in Figure 5c shows an event and fluorescence plotted as ΔF over time in Figure 5i, dynamics plotted in Figure 5m). This classification was done since Docking and Undocking events were limited to the selected ROI for at least 40 frames (Figure 5o) compared to visiting events which moved in and out, along the XY plane more frequently during their residence at the plasma membrane (Figure 5p).

The events of CD63+c classified based on the criteria mentioned above (a‐d) were quantified to determine the density of individual events in the plasma membrane (Figure 5e). The density of docking events (0.041 µm^−2^) was similar to that of undocking events (0.045 µm^−2^). Also, the density of Docking and Undocking events (0.046 µm^−2^) was similar to that of individual docking events and undocking events identified. The number of docking events and undocking events were in equilibrium whereas the density of visiting events (0.210 µm^−2^) was significantly higher than all these events (Figure 5e). For a better representation of this, individual events were plotted by their residence times (Figure 5f). Overall, the density and the residence of docking events and undocking events are always maintained in equilibrium allowing the total number of MVBs available at the plasma membrane for release to be constant during limited exocytosis. The visiting events were found to be higher in number and lower in residence and none of these undergo exocytosis.

### 3.6 MVBs and ISGs are reduced in type‐2 diabetes

**FIGURE 6:**
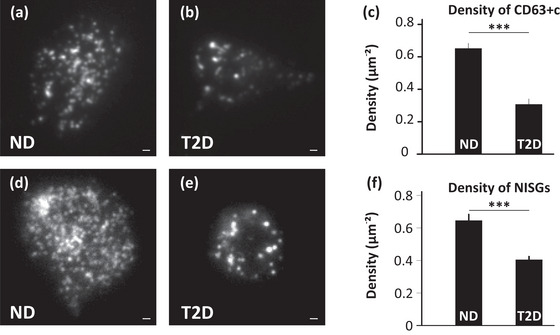
Comparison of MVBs and ISGs distribution in non‐diabetic (ND) and type‐2 diabetic (T2D) β‐cells from human pancreas. a‐b TIRF Image showing CD63+c (mCherry‐CD63) in (a) ND and (b) T2D human islet cells. Scale bar 1 µm. C Density of CD63+c (mCherry‐CD63) in ND and T2D human islet cells. Data are presented as mean ± SEM. For *n* = 27 (2 ND donors), *n* = 16 (three T2D donors) ***p < 0.001. (d,e) Same as (a and b) for NISGs. Scale bar 1 µm. (f) Density of NISGs (NPY‐mCherry) in ND and T2D human islets. Data are presented as mean ± SEM. For *n* = 28 (three ND donors), *n* = 84 (three T2D donors). ***p < 0.001.

Extrapolation of the density of vesicle types observed in the β‐cell line to the human pancreatic islets is more relevant to study the alterations during disorders such as T2D. The density of MVBs and ISGs in pancreatic islet β‐cells of human donors with non‐diabetic (ND) and T2D conditions were evaluated for this purpose. ND and T2D pancreatic islet β‐cells were transduced with mCherry‐CD63 or NPY‐mCherry to label CD63+c or NISGs, respectively. mCherry‐CD63 positive puncta (Figure 6a,b) or NPY‐mCherry positive puncta (Figure 6d,e) were observed in human islet cells. The fluorescent puncta observed in both cases were quantified using an Image J plugin “find maxima” (details in the methods). The density of vesicles in each case was calculated by normalizing the count to the area of that particular islet cell.

In the ND condition, a higher number of mCherry‐CD63 labelled compartments (Figure 6a), but a lesser number in the case of T2D (Figure 6b) was observed. When quantified, a significant decrease in the density of mCherry‐CD63 labelled compartments was seen in T2D cells (0.306 µm^−2^) when compared to ND cells (0.652 µm^−2^) *p* < 0.001 (Figure 6c). Similarly, NPY‐mCherry labelled ISGs were observed in ND human islet cells (Figure 6d) and visually the number was decreased in the case of T2D (Figure 6e). Quantifying the same in many such cells showed NPY‐mCherry labelled ISGs were significantly decreased (*p* < 0.001) in T2D (0.405 µm^−2^) when compared to ND condition (0.648 µm^−2^) (Figure 6f). It was also observed that type‐2 diabetic β‐cells were smaller than their non‐diabetic counterparts. The decrease in density of MVBs and ISGs in diabetic human islet cells is maybe because of their non‐functionality. This might reflect decreased synthesis and secretion from ISGs (Gandasi et al., 2018) or MVBs (Ribeiro et al., 2017, 2023) as shown previously.

## 4 DISCUSSION

Pancreatic β‐cells contain Insulin secretory granules (ISGs) that secrete insulin to maintain glucose homeostasis. The secretory granules are ∼300 nm in diameter, and a single pancreatic β‐cell consists of more than 1000 ISGs (Dean, 1973). NPY, a marker of large dense core vesicles has been used here to label ISGs of pancreatic β‐cells (Gandasi & Barg, 2014). Pancreatic β‐cells synthesize and secrete exosomes, in addition to insulin secretion. Tetraspanin CD63 enriched on intraluminal vesicles (ILVs) or exosomes is well established as a marker of MVBs in other non‐secretory cell types (Andreu & Yáñez‐Mó, 2014). The use of fluorescently labelled CD63 to study MVB exocytosis has not been characterized in pancreatic β‐cells, a secretory cell type where the populations and function of both MVBs and secretory large dense core vesicles such as ISGs might vary compared to a non‐secretory cell. In non‐secretory cells like HeLa cells, HUVEC cells, HT1080 fibrosarcoma cells and A549 cells, CD63 tagged with EGFP, pHluorin, pHluo_M153 or pHuji has been exploited to label MVBs containing intraluminal vesicles (Mahmood et al., 2023; Sung et al., 2020; Verweij et al., 2018). A dual‐color reporter pHluo_M153R‐CD63‐mScarlet has been reported to monitor MVB trafficking before fusion as well as exosome endocytosis in HT1080 fibrosarcoma cells (Sung et al., 2020). There are studies indicating that small EVs (exosomes and microvesicles) also originate from small membrane buds with lesser release of EVs from MVBs (Fan et al., 2023; Fordjour et al., 2022; Tognoli et al., 2023). These vesicles that buds from plasma membrane are labeled using CD9, whereas CD63 mostly does not label these vesicles (Fan et al., 2023; Han et al., 2021; Kowal et al., 2016; Mathieu et al., 2021). Exosome release might not occur majorly through direct budding from the plasma membrane, based on recent studies utilizing CD63 for studying MVBs kinetics (Bai et al., 2021; Bebelman et al., 2020; Liu et al., 2023; Mahmood et al., 2023; Mathieu et al., 2021; Messenger et al., 2018; Ostrowski et al., 2010; Sung et al., 2020; Verweij et al., 2018, 2019). Studies have shown fluorescently labelled CD63 puncta near the plasma membrane similar to what was observed in our results (Figure 1e) (Mahmood et al., 2023; Sung et al., 2020; Verweij et al., 2018). Similar labelling techniques have been employed here to study MVB exocytosis in pancreatic β‐cells. Our study uses tetraspanin CD63 as a marker of MVBs and NPY as a marker of ISGs and showed that NISGs are highly populated vesicles in pancreatic β‐cells when compared to CD63+ compartments (Figure 1i). This study also successfully employed CD63 to visualize CD63+c of pancreatic β‐cells isolated from human islets (Figure 6a,b). This will pave the way for understanding the exosome secretion in single human pancreatic β‐cells and its relation to human diabetes.

**FIGURE 7:**
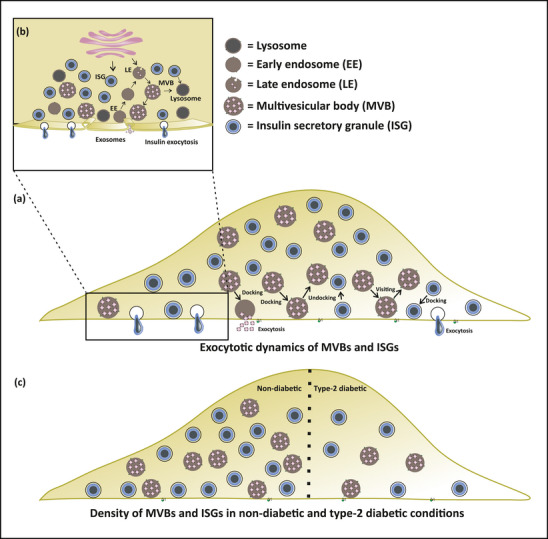
Schematic representation of MVB dynamics and trafficking leading to fusion and exocytosis. (a) The docking, undocking, and visiting dynamics of MVB at the plasma membrane. (b) Detailed representation of a part of the cell showing localization of MVBs with other vesicle types. (c) Density of MVBs and ISGs in non‐diabetic (ND) and type‐2 diabetic (T2D) conditions.

Early endosomes that bud from the plasma membrane either recycle back the proteins to the membrane or mature into late endosomes. These late endosomes acquire intraluminal vesicles (ILVs) to become MVBs (Hessvik & Llorente, 2018; Raposo & Stoorvogel, 2013). MVBs can either fuse with plasma membrane to release exosomes or fuse with lysosomes (Pols & Klumperman, 2009). In this study, no major colocalization of CD63 was found with the markers of other vesicle types like early endosomes, lysosomes and large dense core vesicles (Figures 2, 7). In other cell types like A549, the CD63 fusion events were predominantly MVB fusion events and showed no correlation with lysosomal fusion events (Mahmood et al., 2023). These findings give us the advantage of using CD63 to mainly label MVBs in pancreatic β‐cells.

ISGs are trafficked to the plasma membrane for fusion and exocytosis (Bratanova‐Tochkova et al., 2002; Rorsman & Renström, 2003). Exocytosis of insulin secretory granules would require the trafficking of these granules to the release site, docking of the vesicles to the plasma membrane, followed by priming (Gandasi & Barg, 2014; Olofsson et al., 2002; Omar‐Hmeadi & Idevall‐Hagren, 2021). During SNARE‐complex mediated exocytosis of ISGs, there is a fusion of VAMP2 (v‐SNARE) with t‐SNAREs syntaxin‐1A and SNAP‐25 in the plasma membrane (Omar‐Hmeadi & Idevall‐Hagren, 2021). The detailed mechanism of ISG secretion, exocytotic machinery, including accessory proteins involved, and the dynamics of ISG exocytosis is well studied and understood, but the same remains elusive in the case of MVB secretion. Trafficking and fusion of the MVBs to the plasma membrane are mediated by factors such as Rab GTPases and SNARE complexes (Hessvik & Llorente, 2018; Kowal et al., 2014; Xu et al., 2022). SNAREs like VAMP7 in the human leukemic cell line (K562), YKT6 in human embryonic kidney cells (HEK293) and human lung cancer cells (A549) were found to be the vesicular SNAREs involved in fusion. Target membrane SNAREs (t‐SNAREs) like syntaxin‐4 and SNAP‐23 in Hela cells and syntaxin‐1A in drosophila S2 cells were implied for the fusion and secretion of MVBs (Hessvik & Llorente, 2018; Verweij et al., 2018; Xu et al., 2022). The nature of the SNARE complex involved in the secretion in β‐cells is still not well understood. The detailed studies on the fusion machinery, including the accessory proteins, mechanism and dynamics of MVB secretion need to be explored. There is high importance to exploring the mechanism and dynamics of MVB secretion in secretory cell types like β‐cells.

This study compares and correlates ISG secretion with the MVB secretion dynamics. There could be a high chance of having the same exocytotic machinery for the fusion of both vesicle types so that they are energetically favourable. Here, the possibility of MVBs and ISGs to use the same hotspots populated with proteins involved in the fusion machinery during exocytosis was assessed. On the contrary, CD63+c and NISGs were localized apart near the plasma membrane (Figure 2a and f); CD63 does not localize with syntaxin‐1A, which is a part of the fusion machinery for the release of ISGs (Figure 2b). Therefore, the fusion machinery of ISGs and MVBs are different, which leads us to evaluate the potential differences in the kinetics of exocytosis of these two vesicle types.

In our study, the tetraspanin CD63 has been used to analyse the kinetics of MVB exocytosis and the distribution of its release events in a single pancreatic β‐cell in real‐time (Figure 3). On visualizing the exocytosis of CD63+c and comparing it with the kinetics of NISGs in single β‐cells, slower release of CD63+c (>4 s) was observed while NISG exocytosis is rapid (<1 s) (Figure 3j). In Hela cells, the signal duration of the CD63‐pHluorin fusion events was longer (105.55 s) than NPY‐pHluorin reporter (0.85 s) providing more evidence to the fusion kinetics observed in our study (Verweij et al., 2018). Quantification of these events also revealed a lesser number of CD63+c undergoing exocytosis than that of NISGs (Figure 4c). The difference observed was not only limited to the kinetics of release of CD63+c, a difference in the preferential release site spatially arranged in single cells was also observed. Exocytosis of ISGs is generally localized to a small portion of pancreatic β‐cells, referred to as the active region of release, suggesting the polarized arrangement of ISG exocytosis in β‐cells, which was observed here as well (Figure 4b). These preferential release sites might aid in direct secretion into blood vessels (Qian et al., 2000). Although, NISG exocytosis is polarized (Figure 4b), CD63+c are not the same. CD63+c, on the contrary, release exosomes from non‐peripheral regions (Figure 4a). Though ISGs and MVBs are being secreted from the same cell, the notable difference in their exocytosis is remarkable. Such differences in the exocytosis of these vesicle types could be because of different exocytosis machinery involved in their release. Our study shows that the kinetics of MVB exocytosis in pancreatic β‐cells is similar to MVB exocytosis shown in other cited studies (Mahmood et al., 2023; Verweij et al., 2018).

The availability of MVBs near the plasma membrane is critical for exocytosis. The availability of MVBs was assessed by following the cycle of MVBs at the plasma membrane. In A549 cells, the MVBs were found to typically dock to the membrane (docking events lasted for at least 1 s, but generally longer) before fusion and showed temperature‐dependent fusion kinetics with maximum fusion at 37°C (Mahmood et al., 2023). Based on these temperature kinetics, the CD63+c near the plasma membrane were analysed for their characteristic events: Docking, Undocking, Docking and Undocking, and Visiting (details in the results) at 37°C in pancreatic β‐cells (Figure 5,7a). In the case of insulin granule exocytosis, NISGs dock to the plasma membrane and undergo rapid exocytosis. The stably docked NISGs reside at the plasma membrane for more than 25 s (Docking event) (Gandasi & Barg, 2014). Not all the docked ISGs remain immobilized at the plasma membrane. The loosely attached ISGs undock from the plasma membrane (Undocking event with a resident time of >25 s) without undergoing exocytosis (Gandasi & Barg, 2014). A small population of ISGs, constituting for visiting events, resides for less than 25 s, without leading to exocytosis (Gandasi & Barg, 2014). Our data in the case of CD63+c indicate that different granules population of CD63+c follows distinct dynamics as compared to NISGs. The Majority of the CD63+c events are visiting ones (Figure 5e) and they are more dynamic compared to Docking and Undocking events (Figure 5o,p). The docked CD63+c reside at the plasma membrane for more than ∼6 s (Figure 5f). A population of docked CD63+c undocked after residing for more than ∼6 s in the plasma membrane. The number of these docking events and undocking events were almost equal, indicating the maintenance of a constant number of CD63+c near the plasma membrane when there is limited exocytosis. A majority of visiting events were observed near the membrane with average residence time of 2 s, constituting a more dynamic nature of CD63+c (Figure 5f). Previous studies on ISGs showed less population of ISGs (Visitors) has higher mobility with a residence time of 10–25 s (Gandasi & Barg, 2014). Our data shows that MVBs are more dynamic than ISGs. A similar approach was followed by Jaiswal et al. (2002), who used CD63‐GFP as a lysosome marker to study vesicle exocytosis in non‐secretory cells, whereas CD63‐GFP is now recognized as an exosome marker (Jaiswal et al., 2002). In secretory cells such as pancreatic β‐cells the localization of CD63 with lysosomes is less than 10% (Figure 2d) and the release kinetics can vary from non‐secretory cells.

Several studies have implicated the EV‐mediated cross‐talk affecting the β‐cell function and/or viability (Chidester et al., 2020; Salomon et al., 2022; Xiao et al., 2019). Healthy pancreatic β‐cells and their normal functioning are essential for combating hyperglycaemia and insulin resistance seen during T2D development. β‐cells secrete insulin via the exocytosis of ISGs to maintain the blood glucose, but the insulin secretion is affected during T2D, hence affecting the β‐cell function and eventually viability. Islet cell function and viability are maintained due to the secretion of exosomes, which mediate cross‐talk between other pancreatic β‐cells and other islet cells (Chidester et al., 2020). Exosomes secreted from healthy islets have a protective role in the survival and function of the pancreas (Xiao et al., 2019) by increasing insulin content during abnormal glucose tolerance (Sun et al., 2019). A subset of β‐cells with high expression of CD63 shows enhanced insulin secretion. This subset was diminished in the case of T2D (Rubio‐Navarro et al., 2023). Similarly, islets from T2D patients had decreased secretion of EVs due to increased islet amyloids which could be reversed by adding EVs derived from healthy patients in vitro (Ribeiro et al., 2017). These conditions trigger an immune response and hence alter the exosomal load in circulation (Freeman et al., 2018). In agreement with the above studies, a decreased density of CD63+c and NISGs was observed in single islet β‐cells of T2D patients (Figures 6, 7c). This shows that normal functioning at the single cell level leading to EV secretion, maintaining β‐cell viability, and insulin secretion is altered during T2D. These results show the potential of studying EVs for the diagnosis and treatment of T2D, while also providing insights on the synthesis and secretion of exosomes regulating cell‐to‐cell communication.

Uncovering the molecular mechanism behind exosome secretion has a unique edge in establishing MVB markers for studying the health of islet cells and understanding its secretion in relation to large dense core vesicle secretion, such as insulin. This will lay a foundation for using exosomes as diagnostic and prognostic tool for diabetes.

## AUTHOR CONTRIBUTIONS

**Priyadarshini Veerabhadraswamy**: Data curation (lead); formal analysis (lead); investigation (lead); methodology (lead); software (lead); validation (lead); visualization (lead); writing—original draft (lead); writing—review and editing (lead). **Kiran Lata**: Data curation (supporting); formal analysis (supporting); methodology (supporting). **Sristi Dey**: Formal analysis (supporting); visualization (supporting). **Prajakta Belekar**: Data curation (supporting); formal analysis (supporting); visualization (supporting). **Lakshmi Kothegala**: Investigation (supporting); methodology (supporting); resources (supporting); software (supporting); supervision (supporting); validation (supporting); visualization (supporting). **Vidya Mangala Prasad**: Data curation (supporting); investigation (supporting); methodology (supporting); resources (supporting); software (supporting); supervision (supporting); validation (supporting); writing—review and editing (supporting). **Nikhil R. Gandasi**: Conceptualization (lead); data curation (lead); formal analysis (lead); funding acquisition (lead); investigation (lead); methodology (lead); project administration (lead); resources (lead); software (lead); supervision (lead); validation (lead); visualization (lead); writing—original draft (lead); writing—review and editing (lead).

## INSTITUTIONAL REVIEW BOARD STATEMENT

Human islets were provided through the JDRF award 19‐DSA‐048 (ECIT Islet for Basic Research Program) and the Alberta Diabetes Institute Islet‐Core, Canada and Nordic Network for Clinical Islet Transplantation (Uppsala), Sweden. Human islets are being utilized in the University of Gothenburg, Sweden and Indian Institute of Science, India as per ethics protocols numbered—098‐18 from Regionala Etikprovningsnamnden Goteborg and 08/20 July 2022 from Institutional Human Ethics Committee (IHEC), Indian Institute of Science, India respectively.

## CONFLICT OF INTEREST STATEMENT

The authors declare no conflict of interest.

## Supporting information



Supporting Information

Supporting Information

Supporting Information
