# Supplementary figures and images for: Comparison of localization and release of multivesicular bodies and secretory granules in islet cells: Dysregulation during type‐2 diabetes
Source: J Extracell Biol. 2024 Nov 29;3(11):e70014. doi: 10.1002/jex2.70014 (PMC11605659; doi:10.1002/jex2.70014)

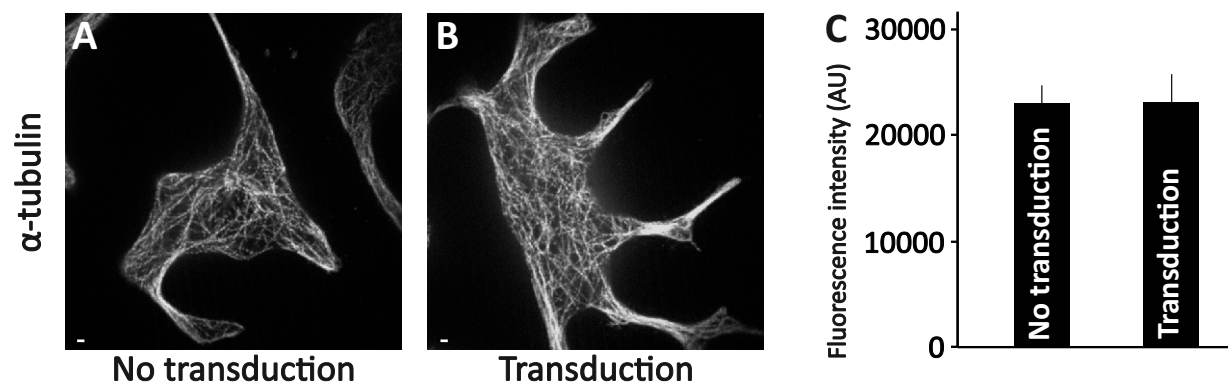

Figure S1

Supplement: Supplementary file 1 — Supporting Information [file JEX2-3-e70014-s001.pdf]

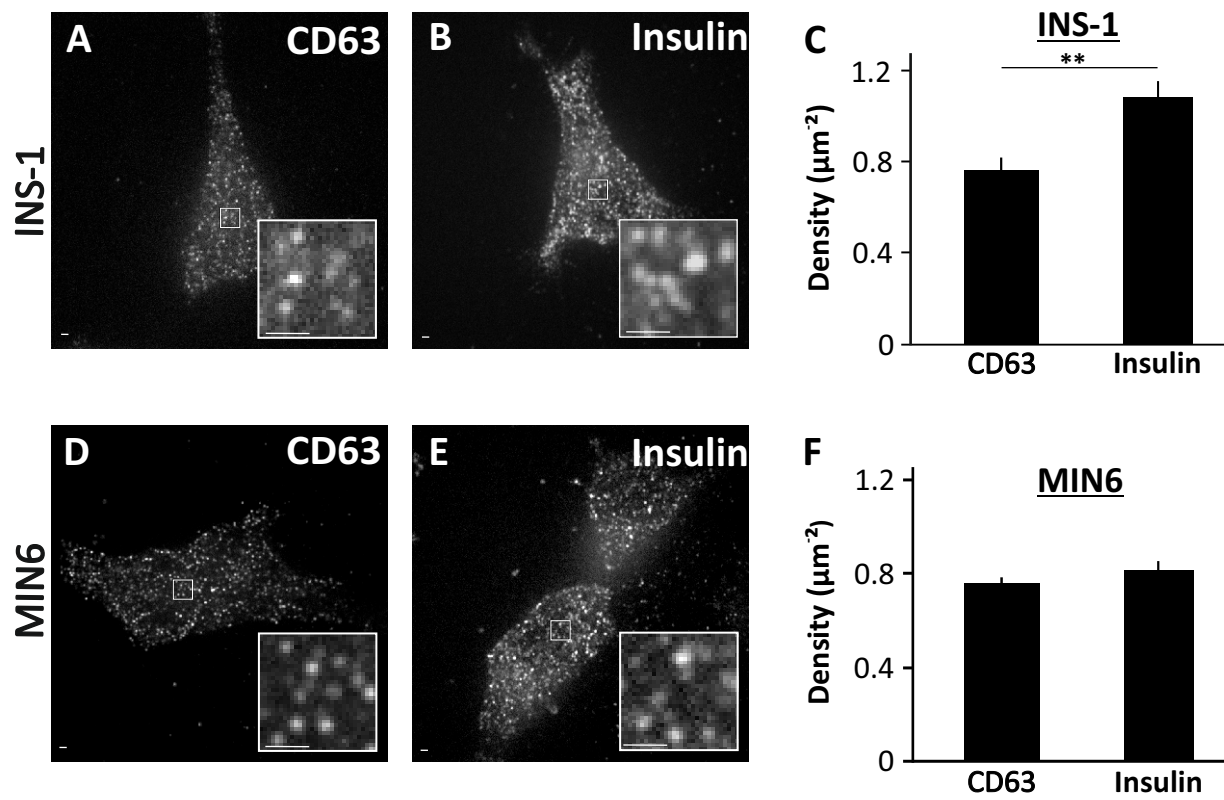

Figure S2

Supplement: Supplementary file 2 — Supporting Information [file JEX2-3-e70014-s003.pdf]
