# Supplementary material for: Comparison of localization and release of multivesicular bodies and secretory granules in islet cells: Dysregulation during type‐2 diabetes
Source: J Extracell Biol. 2024 Nov 29;3(11):e70014. doi: 10.1002/jex2.70014 (PMC11605659; doi:10.1002/jex2.70014)
Supplement: Supplementary file 3 — Supporting Information [file JEX2-3-e70014-s002.docx]

**Supplementary information**

**Figure legends (Supplementary)**

Figure S1. **Fluorescence intensity of α-tubulin during adenovirus transduction.**

**A** Immunofluorescent image of INS-1 832/13 cell for α-tubulin without adenovirus transduction. Scale bar 1 μm.

**B** Immunofluorescent image of INS-1 832/13 cell for α-tubulin with adenovirus transduction of mCherry-CD63. Scale bar 1μm.

**C** Fluorescence intensity of α-tubulin in INS-1 832/13 cells without transduction and with adenovirus transduction of mCherry-CD63 for images like **A-B**. Data represented as mean ± SEM for n=20 cells for no transduction and adenovirus transduction from at least 2 independent experiments in each case.

Figure S2. **Density of insulin secretory granules (ISGs) and multivesicular bodies (MVBs) in two different pancreatic β-cell lines.**

**A-B** Immunofluorescent images of INS-1 832/13 cells with **(A)** CD63+ compartments and **(B)** Insulin labelled ISGs. Scale bar 1 μm.

**C** Density of CD63+ compartments and Insulin labelled ISGs in INS-1 832/13 cells for images like **A-B**. Data represented as mean ± SEM for n=20 cells for CD63 or Insulin density from at least 2 independent experiments in each case. **p < 0.01.

**D-E** Immunofluorescent images of MIN6 cells with **(D)** CD63+ compartments and **(E)** Insulin labelled ISGs. Scale bar 1 μm.

**F** Density of CD63+ compartments and Insulin labelled ISGs in MIN6 cells for images like **D-E**. Data represented as mean ± SEM for n=20 cells for CD63 or Insulin density from at least 2 independent experiments in each case.
